# Effects of transmembrane protein 106B genetic variations on disease progression in Parkinson’s disease

**DOI:** 10.3389/fnagi.2026.1873947

**Published:** 2026-07-23

**Authors:** Wanbing Zhao, Xiaoniu Liang, Bolin Hu, Wenbo Yu, Jianjun Wu, Yimin Sun, Jian Wang, Yun Fan

**Affiliations:** 1 Department of Neurology and National Research Center for Aging and Medicine and National Center for Neurological Disorders, State Key Laboratory of Medical Neurobiology, Huashan Hospital, Fudan University, Shanghai, China; 2 Department of General Practice, Longgang District Central Hospital of Shenzhen, Shenzhen, China

**Keywords:** transmembrane protein 106B, Parkinson’s disease, genetic variations, rs3173615, disease progression

## Abstract

**Background:**

Transmembrane protein 106B (*TMEM106B*) variations not only act as genetic modifiers of the risk of developing frontotemporal lobar degeneration but also correlate with the heterogeneity of clinicopathological phenotypes in other neurodegenerative diseases. However, the roles of TMEM106B in Parkinson’s disease (PD) are sparsely explored. This study aims to explore whether *TMEM106B* variants influence the trajectories of clinical phenotypes in PD.

**Methods:**

We longitudinally followed 241 PD patients and genotyped their single nucleotide polymorphism (SNP) of rs3173615. Patients were categorized according to rs3173615 genotypes into GG (major allele homozygotes), GC (heterozygotes), and CC (minor allele homozygotes) groups. All patients completed clinical evaluations and neuropsychological tests at baseline and every follow-up. Linear mixed-effects models were adopted to evaluate the association between rs3173615 genotypes and longitudinal disease progression in PD.

**Results:**

At baseline, both the GG and GC groups presented less severe excessive daytime sleepiness than the CC group, and no association between the remaining clinical characteristics and the genotypes of rs3173615 was observed among the three groups. Longitudinally, compared with the CC group, the GC group manifested a significantly faster exacerbation of depression, visuospatial function and quality of life (QoL), and the GG group showed the same tendency as the GC group, though without statistical significance.

**Conclusion:**

*TMEM106B* rs3173615 is a genetic modifier for the trajectory of depression, visuospatial function and QoL in PD. Our findings suggest the potential involvement of TMEM106B in the pathogenesis of disease progression in PD, especially in the deterioration of depression, cognition and QoL.

## Introduction

Variants of transmembrane protein 106B (*TMEM106B*) were first found to be genetic modifiers of risk for frontotemporal lobar degeneration (FTLD) with TDP-43 pathology (FTLD-TDP) in a genome-wide association study in 2010 (Van Deerlin et al., 2010). Similar results were replicated in another FTLD cohort from Flanders, Belgium (van der Zee et al., 2011). Furthermore, the major allele of *TMEM106B* single nucleotide polymorphism (SNP) rs1990622 was also found to correlate with earlier age at onset (Cruchaga et al., 2011), faster decrease in cognitive function (Tropea et al., 2019), and higher risk of developing neuroastroglial tauopathy in FTLD patients (Llibre-Guerra et al., 2021). In addition to its roles in FTLD, the major allele of *TMEM106B* SNP rs1990622 also increases the risk of developing Alzheimer’s disease (AD) (Hu et al., 2021), the most common neurodegenerative diseases (NDs), whereas its minor allele decreases the risk of developing hippocampal sclerosis in AD (Rutherford et al., 2012). Interestingly, another study reported that AD risk is remarkably influenced by the interaction of APOE with rs1595014, another SNP in *TMEM106B* (Jun et al., 2016). Studies also revealed that multiple SNPs of *TMEM106B* were correlated with the clinical and pathological phenotypes of amyotrophic lateral sclerosis (ALS) (Vass et al., 2011; Mao et al., 2021) and hippocampal sclerosis of aging (Yu et al., 2015). However, the effects of *TMEM106B* genetic variations on Parkinson’s disease (PD), the second most common NDs after AD, were poorly investigated.

Given the findings about the influences of *TMEM106B* variants on the risk and phenotypes of NDs, especially FTLD, from clinical studies, the physiological function of TMEM106B and its pathogenic mechanisms in FTLD have received considerable attention and have been well studied over the past decade. Studies suggest that TMEM106B is an integral type II transmembrane lysosomal protein and plays vital roles in lysosomal morphology, localization, acidification, anterograde and retrograde trafficking, and function (Brady et al., 2013; Schwenk et al., 2014). The expression of TMEM106B mRNA and protein was dysregulated in FTLD, AD and ALS (Van Deerlin et al., 2010; Mao et al., 2021; Satoh et al., 2014). Both overexpression and knockdown/knockout of *TMEM106B* resulted in lysosomal dysfunctions which were proved to be a common and critical pathological process in the development and progression of NDs (Brady et al., 2013; Schwenk et al., 2014). Therefore, TMEM106B was thought to participate in the pathogenesis of NDs through influencing lysosomal function. Notably, similar to other pathogenic amyloid proteins (such as amyloid *β*, Tau, and *α*-synuclein) in NDs, TMEM106B was found to form amyloid fibrils in brains of patients with NDs, including PD (Schweighauser et al., 2022; Chang et al., 2022; Fan et al., 2022). This surprising finding expands our perspective on the roles that TMEM106B plays in the pathogenesis of NDs. Moreover, unlike disease-associated conformational polymorphisms of *α*-synuclein (*α*-syn) in *α*-synucleinopathies or Tau in tauopathies, the conformation of TMEM106B fibrils is not influenced by neurodegenerative disease conditions but is instead determined by the genotypes of *TMEM106B* rs3173615 (Fan et al., 2023), the only one SNP located in exonal region, which results in the nonsynonymous mutation p.Thr185Ser in TMEM106B. The causal relationship between *TMEM106B* rs3173615 haplotype and TMEM106B fibrils structural polymorphism in NDs further suggests the involvement of TMEM106B in the pathogenesis of NDs.

As mentioned above, previous explorations about TMEM106B involvement in NDs were mostly focused on FTLD, followed by AD, with PD sparsely studied. Taking the indiscriminate amyloid fibrils composed of TMEM106B formation in FTLD, AD and PD, it is plausible to speculate that TMEM106B roles in PD is also nonnegligible. Nonetheless, to our knowledge, only one longitudinal study to date has investigated the *TMEM106B* effect on PD clinical phenotypes, and found that the major allele of *TMEM106B* rs1990622 was associated with the accelerated cognition decline in PD patients (Tropea et al., 2019). However, this study only assessed the global cognitive function without elevating specific cognitive subdomains and other non-motor symptoms (NMS). Thus, in combination with the effects of *TMEM106B* rs3173615 haplotype on the clinicopathological phenotypes across multiple NDs and its deterministic role in TMEM106B fibril conformation polymorphisms (Fan et al., 2023), this study aims to systematically investigate the effects of *TMEM106B* genetic variation, SNP rs3173615, on clinical phenotypes in PD patients, which will provide potential new clues for the participation of TMEM106B in PD.

## Materials and methods

### Subjects

All subjects were successively enrolled at Huashan Hospital, Fudan University from February 2014 to December 2020. The diagnosis of PD was made by two neurologists specializing in movement disorder according to the United Kingdom Brain Bank criteria (Hughes et al., 1992) (for subjects enrolled before 2016) and MDS Clinical Diagnostic Criteria published in 2015 (Postuma et al., 2015) (for subjects enrolled after 2016). Recruitment criteria included: (1) age≥50 years; (2) no family history of PD; (3) no other identified neurological diseases, such as stroke, dementia, and brain trauma; (4) no malignant diseases, including tumors, or heart failure; (5) completion of at least two research visits, spaced a minimum of 10 months apart; (6) completion of genetic testing. Subjects with parkinsonism induced by identified factors, such as drug-induced parkinsonism, and vascular parkinsonism, were excluded. All subjects were annually followed up.

### Standard protocol approvals, registrations, and patient consents

Approvement from the Human Studies Institutional Review Board, Huashan Hospital, Fudan University was acquired in this study. Informed consents in conformity to the Declaration of Helsinki were obtained from all participants.

### Clinical assessments

All clinical assessments and neuropsychological tests at baseline and at each subsequent visit were completed by two trained physicians. Motor function was measured by Hoehn & Yahr (H&Y) grading and the Unified Parkinson’s Disease Rating Scale-Part III (UPDRS-III) during the anti-parkinsonian medications-OFF state. NMS were assessed by Non-Motor Symptoms Scale (NMSS) (Chaudhuri et al., 2007). Besides, some common NMS were further evaluated by some corresponding scales and questionnaires. Depression was assessed by Geriatric Depression Rating Scale (GDS) (Ertan et al., 2005) and Beck Depression Inventory (BDI) (Richter et al., 1998). Two kinds of sleeping dysfunctions, rapid-eye-movement (REM)-sleep behavior disorder (RBD) and excessive daytime sleepiness (EDS), were measured by REM-sleep Behavior Disorder Screening Questionnaire (RBDSQ) (Wang et al., 2015) and Epworth Sleepiness Scale (ESS) (Chen et al., 2002), respectively. Olfactory function was detected by Sniffin’ Sticks screening 12 test (SSST-12) (Hummel et al., 2001). Quality of life (QoL) was recorded by the 39-item Parkinson’s Disease Questionnaire (PDQ-39) (Tsang et al., 2002). To standardize the medications data, the dosage of anti-parkinsonian medications was calculated into the levodopa equivalent daily dose (LED) as previous research performed (Tomlinson et al., 2010).

All subjects were in the medications-ON condition when they conducted cognitive function tests. Global cognitive functions were detected by the Mini Mental State Examination (MMSE) (Katzman et al., 1988). In addition, five specific cognitive domains were assessed by a series of neuropsychological tests. These tests included: Symbol Digit Modalities Test (SDMT) (Sheridan et al., 2006) and Trail Making Test A (TMT-A) (Zhao et al., 2013) for attention and working memory; Stroop Color-Word Test (CWT) (Steinberg et al., 2005) and Trail Making Test B (TMT-B) (Zhao et al., 2013) for executive function; Boston Naming Test (BNT) (Goldman et al., 2015) and Animal Fluency Test (AFT) (Lucas et al., 2005) for language; Auditory Verbal Learning Test (AVLT) (Guo et al., 2009) and delayed recall of Rey-Osterrieth Complex Figure Test (CFT- delay) (Caffarra et al., 2002) for memory; Clock Drawing Test (CDT) (Guo et al., 2008) and copy task of Rey-Osterrieth Complex Figure test (CFT) (Caffarra et al., 2002) for visuospatial function.

### Genotyping

Blood samples were collected from all participants at enrollment. Genomic DNA extracted from blood samples was used for genotyping arrays. The Illumina Infinium Asian Screening Array that covers approximately 700,000 SNPs was adopted in this study via a standard protocol. This study utilized the 1,000 Genomes Project Phase 3 Han Chinese in Beijing/Southern Han Chinese (CHB/CHS) database as the reference panel, and performed genotype imputation using IMPUTE5 (https://jmarchini.org/impute5/). The *TMEM106B* SNP rs3173615 was extracted from the imputed dataset for subsequent analysis. Participants were categorized into three genotype groups: GG (major allele homozygotes), GC (heterozygotes), and CC (minor allele homozygotes). Post-imputation filtering was conducted to retain only SNP loci with IMPUTE-info (imputation information score, info) quality scores > 0.7. The genotype distribution of rs3173615 was tested for Hardy–Weinberg equilibrium (HWE) using the chi-square test.

### Statistical analysis

Categorical data were shown in the form of frequencies (%), and quantitative data were expressed as mean ± standard deviation (SD) or median (interquartile range). Homogeneity of variance test was performed for all quantitative variables. Among the three genotype groups, one-way analysis of variance or Kruskal-Wallis test was used for comparing normally and non-normally distributed quantitative variables, respectively; and chi-squared test was performed to compare the categorical variables. The association of *TMEM106B* SNP rs3173615 and clinical characteristics at baseline was analyzed using generalized linear model adjusted with age, sex, education, disease duration, and LED.

Linear mixed-effects models (LMM) were used to assess the associations between rs3173615 genotypes and clinical outcomes over disease duration (years), with covariates including sex, baseline age, education, and LED. Random intercepts and slopes for disease duration were included at the subject level to account for individual variability. Residual analyses were performed to verify model assumptions. The rs3173615 variant was first analyzed under a codominant genetic model, treating GG, GC, and CC as separate genotype categories, which was prespecified as the primary analysis. Additional genetic models, including dominant, recessive, overdominant, and additive models, were further evaluated for the clinical phenotypes showing evidence of association in the primary codominant model, to further explore potential genetic effect patterns. Two-tailed *p* < 0.05 was considered statistically significant. Statistical significance was indicated using asterisk notation (*p < 0.05, **p < 0.01, ***p < 0.001, and ****p < 0.0001). Data analysis was conducted using SAS 9.4 (SAS Institute Inc., Cary, NC, United States) and figures were generated using R Software (version 4.1.2).

## Results

### PD cohort

**Figure 1:**
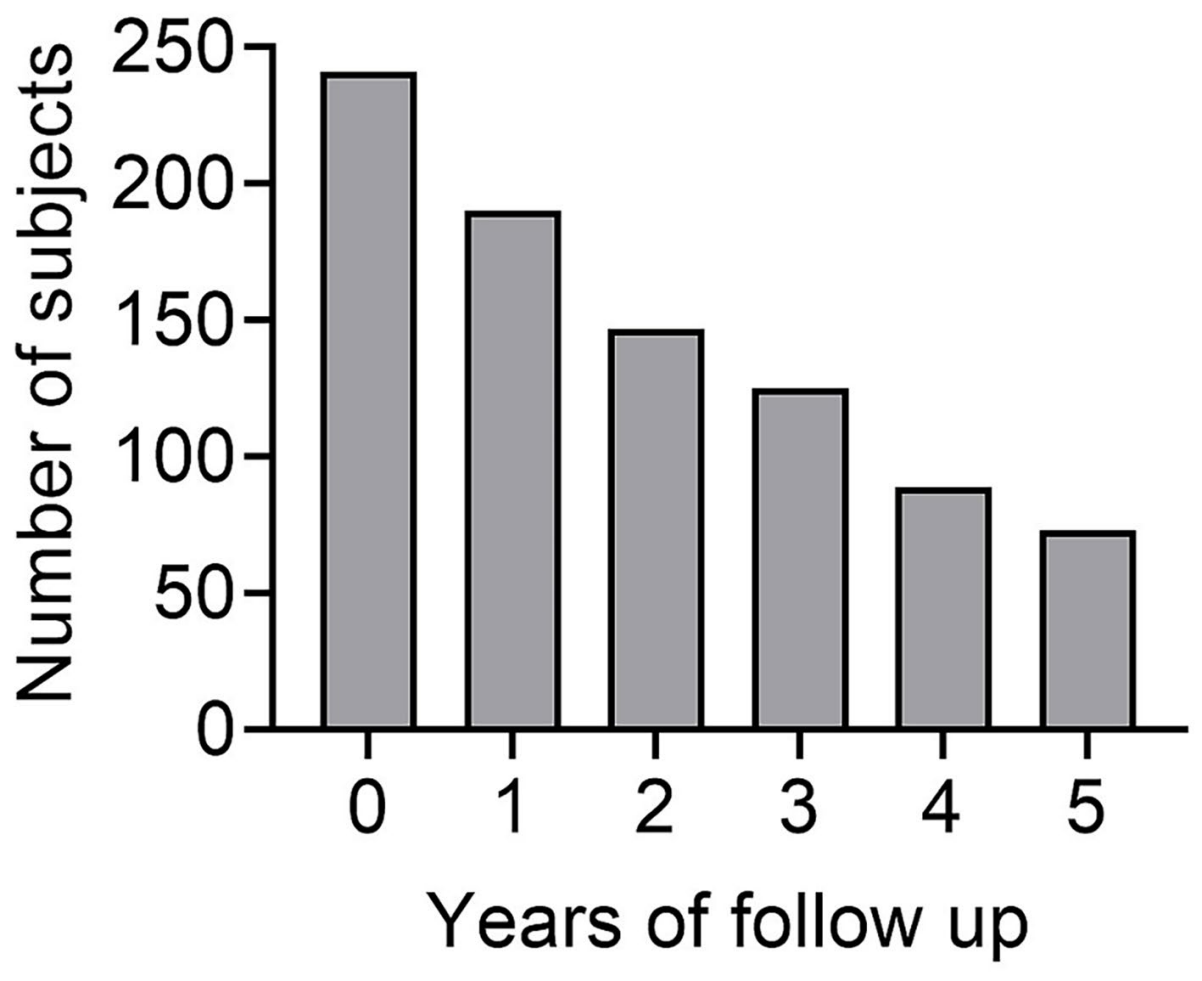
Number of PD patients followed at each year. The figure shows the number of PD patients at each follow-up time point during the longitudinal study.

A total of 241 PD patients (148 males, 93 females), with a mean (SD) age of 63.36 (5.86) years and a mean (SD) disease duration of 60.59 (53.37) months, were recruited in this cohort. The demographic and clinical characteristics of the PD patients at baseline, including motor symptoms, NMS and cognitive function, were shown in Table 1. The only coding SNP identified in *TMEM106B*, rs3173615, was detected and analyzed. The PD patients were followed for up to 5 years with a mean (SD) follow-up of 3.99 (1.12) years. The number of PD patients followed up at each year was showed in Figure 1. Motor symptoms, NMS and cognitive function were assessed for each PD patient at yearly follow-ups.

**Table 1:** Demographic and clinical characteristics of PD patients at baseline.

Variables	*N* = 241
Sex (M, %)	148 (61.41)
Age (y)	63.36 ± 5.86
Education (y)	10.95 ± 4.24
BMI (kg/m^2^)	22.89 ± 2.88
AAO (y)	58.28 ± 6.11
Disease duration (M)	60.59 ± 53.57
LED (mg/d)	611.46 ± 332.80
PDQ-39	38.86 ± 28.34
Motor symptoms and signs	
H&Y	2 (1, 3)
UPDRS-III	30.25 ± 16.89
Non-motor symptoms and signs	
GDS	11.77 ± 7.05
BDI	13.41 ± 9.75
NMSS	11.76 ± 6.34
ESS	6.83 ± 4.76
RBDSQ	4.56 ± 3.33
SSST-12	4.43 ± 2.43
Cognitive function	
MMSE	26.37 ± 3.80
AVLT-delay recall	3.74 ± 2.33
AVLT-T	21.55 ± 8.80
CFT-delay recall	12.06 ± 7.45
CWT-C time(s)	89.79 ± 30.08
CWT-C right	44.66 ± 6.03
TMT-B(s)	187.09 ± 81.43
BNT	21.99 ± 4.96
AFT	15.02 ± 4.24
CFT	28.79 ± 9.04
CDT	19.33 ± 7.58
SDMT	26.69 ± 12.45
TMT-A(s)	83.15 ± 52.20

AAO, age at onset; AFT, Animal Fluency Test; AVLT, Auditory Verbal Learning Test; BDI, Beck Depression Inventory; BMI, body mass index; BNT, Boston Naming Test; CDT, Clock Drawing Test; CFT, Rey-Osterrieth Complex Figure Test; CWT, Stroop Color-Word Test; ESS, Epworth Sleepiness Scale; GDS, Geriatric Depression Rating Scale; H&Y, Hoehn and Yahr; LED, levodopa-equivalent daily dose; MMSE, Mini Mental State Examination; NMSS, Non-Motor Symptoms Scale; RBDSQ, Rapid-Eye-Movement Sleep Behavior Disorder Screening Questionnaire; SSST-12, Sniffin’ Sticks screening 12 test; SDMT, Symbol Digit Modality Test; TMT, Trail Making Test; UPDRS-III, Unified Parkinson’s Disease Rating Scale part III. The data of H&Y is presented as median (25, 75%), and the other continuous data are presented as mean ± SD.

### Characteristics of PD patients with different genotypes of *TMEM106B* at baseline

PD patients were classified into the GG (major allele homozygotes), GC (heterozygotes) and CC (minor allele homozygotes) groups according to rs3173615 genotypes. In detail, 89, 121 and 31 PD patients were categorized into the GG, GC and CC groups, respectively, and the clinical phenotypes of PD patients at baseline were compared among the three groups (Table 2). Of note, the CC group exhibited more severe EDS assessed by ESS, as well as the worst language function assessed by BNT compared with the GG and GC groups at baseline. The remaining demographic and clinical features among the three groups showed no distinction.

**Table 2:** Demographic and clinical characteristics of PD patients with different genotypes of *TMEM106B* rs3173615 at baseline.

Variables	GG (*N* = 89)	GC (*N* = 121)	CC (*N* = 31)	*p*-value
Sex (M, %)	53 (59.55)	75 (61.98)	20 (64.52)	0.8725
Age (y)	62.82 ± 5.43	63.75 ± 6.22	63.35 ± 5.70	0.2514^#^
Education (y)	11.22 ± 4.46	10.78 ± 4.04	10.84 ± 4.44	0.5011
BMI (kg/m^2^)	22.77 ± 2.88	23.13 ± 2.89	22.29 ± 2.82	0.9721^#^
AAO (y)	57.81 ± 5.87	58.62 ± 6.33	58.32 ± 6.05	0.7130
Disease duration (M)	59.56 ± 53.50	61.32 ± 55.11	60.46 ± 48.55	0.9550
LED (mg/d)	541.38 ± 324.53	656.31 ± 341.14	622.81 ± 310.79	0.1156
PDQ-39	38.06 ± 28.29	39.44 ± 28.55	38.77 ± 28.78	0.9471
Motor symptoms and signs				
H&Y	2 (1, 3)	2 (1, 3)	2 (1, 3)	0.7891
UPDRS-III	29.29 ± 17.61	30.66 ± 16.50	31.24 ± 16.89	0.7660
Non-motor symptoms and signs				
GDS	10.97 ± 6.70	12.31 ± 7.14	11.83 ± 7.77	0.5723
BDI	12.61 ± 9.04	13.73 ± 9.58	14.50 ± 12.52	0.7428
NMSS	12.04 ± 6.18	11.65 ± 6.69	11.33 ± 5.43	0.8694
ESS	6.59 ± 4.71	6.43 ± 4.44	9.41 ± 5.70	0.0464*
RBDSQ	4.76 ± 3.39	4.37 ± 3.30	4.71 ± 3.35	0.7406
SSST-12	4.70 ± 2.46	4.28 ± 2.45	4.39 ± 2.25	0.5593
Cognitive function				
MMSE	25.92 ± 4.61	26.82 ± 2.79	25.64 ± 4.90	0.9273
AVLT-delay recall	4.10 ± 2.26	3.64 ± 2.30	3.14 ± 2.61	0.2303
AVLT-T	22.90 ± 8.48	21.38 ± 8.50	18.43 ± 10.53	0.4321^#^
CFT-delay recall	13.03 ± 8.09	12.07 ± 7.32	9.19 ± 5.44	0.1000^#^
CWT-C time(s)	90.28 ± 29.21	87.91 ± 29.81	97.40 ± 33.93	0.3763
CWT-C right	44.86 ± 5.20	44.67 ± 6.57	44.05 ± 5.76	0.8688
TMT-B(s)	180.47 ± 96.58	189.63 ± 69.44	193.95 ± 88.15	0.1338
BNT	23.05 ± 4.43	21.80 ± 4.76	20.00 ± 6.47	0.0394*
AFT	15.43 ± 4.34	14.95 ± 4.11	14.14 ± 4.59	0.3655
CFT	29.64 ± 8.97	28.68 ± 8.72	27.00 ± 10.72	0.2739
CDT	19.84 ± 7.06	19.82 ± 7.55	16.15 ± 8.41	0.1314
SDMT	28.60 ± 12.30	26.71 ± 12.53	21.59 ± 11.59	0.8497^#^
TMT-A(s)	78.03 ± 62.20	85.58 ± 45.25	86.52 ± 52.39	0.0700

AAO, age at onset; AFT, Animal Fluency Test; AVLT, Auditory Verbal Learning Test; BDI, Beck Depression Inventory; BMI, body mass index; BNT, Boston Naming Test; CDT, Clock Drawing Test; CFT, Rey-Osterrieth Complex Figure Test; CWT, Stroop Color-Word Test; ESS, Epworth Sleepiness Scale; GDS, Geriatric Depression Rating Scale; H&Y, Hoehn and Yahr; LED, levodopa-equivalent daily dose; MMSE, Mini Mental State Examination; NMSS, Non-Motor Symptoms Scale; PDQ-39, 39-item Parkinson’s disease questionnaire; RBDSQ, Rapid-Eye-Movement Sleep Behavior Disorder Screening Questionnaire; SSST-12, Sniffin’ Sticks screening 12 test; SDMT, Symbol Digit Modality Test; TMT, Trail Making Test; UPDRS-III, Unified Parkinson’s Disease Rating Scale part III. The data of H&Y is presented as median (25, 75%), and the other continuous data are presented as mean ± SD. The categorical variables were compared among the three groups by the chi-squared test. The nonnormal distributed quantitative variables were compared among the three groups by Kruskal-Wallis test. ^#^ The normal distributed quantitative variables were compared among the three groups by one-way analysis of variance. * *p* < 0.05.

### Association of baseline phenotypes and *TMEM106B* genetic variations

To investigate the association between clinical phenotypes of PD patients at baseline and *TMEM106B* rs3173615 genotypes, the generalized linear model analysis was conducted with sex, age, education, disease duration, and LED at baseline adjusted, using the CC group as the reference group. The results showed that both the GG group (*β* = −3.0934, *p* = 0.0219) and the GC group (*β* = −3.5990, *p* = 0.0047) were less likely to suffer from EDS compared with the CC group (Table 3). The association between other clinical characteristics at baseline and *TMEM106B* rs3173615 genotypes was not found (Supplementary Table S1). Additionally, sensitivity analyses under alternative genetic models were performed for ESS. Consistent with the codominant model, the recessive model (CC vs. GG + GC) showed a significant association with baseline ESS (*β* = 3.41, *p* = 0.0050), while no significant associations were observed under the dominant or overdominant models (Supplementary Table S2).

**Table 3:** Relationship with clinical phenotypes at baseline compared with the CC group.

	GG		GC	
	*β* (95% CI)	*p*-value	*β* (95% CI)	*p*-value
ESS	−3.0934(−5.7310, −0.4559)	0.0219*	−3.5990(−6.0716, −1.1264)	0.0047**

*β*, beta coefficient; CI, confidence interval; ESS, Epworth Sleepiness Score.

*p < 0.05, **p < 0.01.

### Effects of *TMEM106B* genotypes on clinical phenotypes’ trajectory in PD patients

**Figure 2:**
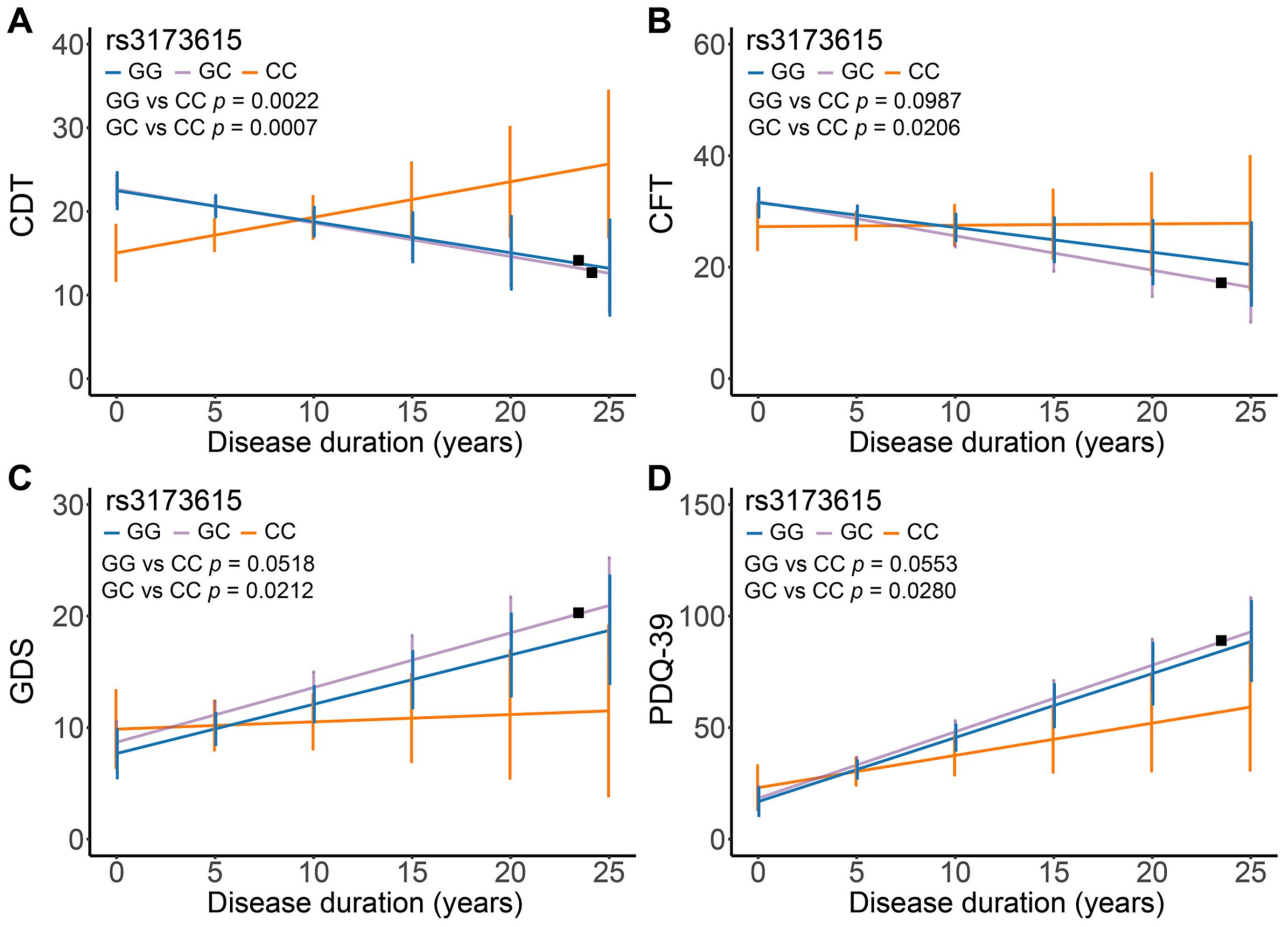
Effects of *TMEM106B* rs3173615 on longitudinal trajectories of clinical phenotypes in PD patients. Longitudinal associations between rs3173615 genotypes and clinical progression were assessed using LMM. Predicted trajectories of clinical scores over disease duration were generated based on the fitted LMM. **(A)** LMM indicates that both the GG and the GC group had faster decrease in CDT scores over time. The GC group showed faster decrease in CFT scores **(B)**, and faster increase in GDS **(C)** and PDQ-39 scores **(D)** over time analyzed by LMM. Genotypes are indicated by color, and bars represent 95% confidence intervals. Exact *p* values shown in the figure represent the significance of differences in longitudinal changes between the GG or GC groups and the CC group (reference group). The black solid square indicates the longitudinal trajectories that are significantly different from the CC group (*p* < 0.05).

LMM was performed to explore the influence of *TMEM106B* rs3173615 genotypes on the longitudinal progression of clinical phenotypes in PD patients. We chose disease duration as the time scale and adjusted for age, sex, education, and LED. Notably, *TMEM106B* rs3173615 genetic variations exerted a remarkable effect on visuospatial function change in PD patients. Specifically, compared with the CC group, both the GG group (*β* = −0.8600, *p* = 0.0022) and the GC group (*β* = −0.9052, *p* = 0.0007) showed a faster decline in CDT. The G allele of rs3173615 was associated with a faster CFT decline in the GC group (*β* = −0.7943, *p* = 0.0206), with the same tendency in the GG group though without statistical significance (*β* = −0.5929, *p* = 0.0987). In addition, SNP rs3173615 had an influence on the changes in depression and QoL in PD patients. In comparison with the CC group, the GC group showed significantly faster depression progress assessed by the GDS (*β* = 0.5367, *p* = 0.0212) and a greater decline in QoL assessed by the PDQ-39 (*β* = 1.7691, *p* = 0.0280). A similar trend was also observed in GG group, although the associations did not reach statistical significance (GDS, *β* = 0.4725, *p* = 0.0518; PDQ-39, *β* = 1.6158, *p* = 0.0553) (Figure 2; Table 4; Supplementary Table S3). No significant associations were observed between rs3173615 genotypes and the longitudinal progression of other clinical phenotypes (Supplementary Table S4). Additionally, sensitivity analyses under alternative genetic models were conducted for the four variables that showed significant longitudinal associations in the primary codominant analysis (Table 4). The recessive model (CC vs. GG + GC) showed significant associations for all outcomes: CDT (*β* = 0.8897, *p* = 0.0005), CFT (*β* = 0.7180, *p* = 0.0295), GDS (*β* = −0.5123, *p* = 0.0219), and PDQ-39 (*β* = −1.7022, *p* = 0.0280). The additive model showed a nominally significant effect for CDT (*β* = 0.2959, *p* = 0.0252), while no significant associations were observed under the dominant or overdominant models (Supplementary Table S5). These findings indicate that rs3173615 genotypes are associated with the longitudinal deterioration of visuospatial function, depression and QoL in PD patients, with the most prominent effects observed in the GC group compared with the CC group.

**Table 4:** Estimates for change in clinical phenotypes trajectory compared with the CC group.

	GG		GC	
	*β* (95% CI)	*p*-value	*β* (95% CI)	*p*-value
CDT	−0.8600 (−1.4045, −0.3155)	0.0022**	−0.9052 (−1.4188, −0.3916)	0.0007***
CFT	−0.5929 (−1.2983, 0.1125)	0.0987	−0.7943 (−1.4647, −0.1239)	0.0206*
GDS	0.4725 (−0.0037, 0.9488)	0.0518	0.5367 (0.0810, 0.9923)	0.0212*
PDQ-39	1.6158 (−0.0370, 3.2687)	0.0553	1.7691 (0.1929, 3.3453)	0.0280*

*β*, beta coefficient; CDT, Clock Drawing Test; CFT, Rey-Osterrieth Complex Figure Test; CI, confidence interval; GDS, Geriatric Depression Rating Scale; PDQ-39, 39-item Parkinson’s disease questionnaire.

*p < 0.05, **p < 0.01, ***p < 0.001.

## Discussion

This study investigated the effects of the *TMEM106B* rs3173615 genotypes on disease progression in PD patients. Firstly, the study compared the baseline clinical phenotypes among the three groups classified by the genotypes of rs3173615. The results demonstrated that the CC group showed more severe EDS and worse language function measured by BNT than the GG and GC groups at baseline, and no significant difference in the remaining clinical phenotypes at baseline was found among the three groups. Secondly, the association between the baseline clinical phenotypes and *TMEM106B* genotypes was determined. And the result found that compared with the CC group, both the GG and GC groups showed lower ESS scores, indicating less severe EDS. Finally, the effects of rs3173615 on disease progression in PD were revealed. It was found that rs3173615 exerted effects on the progression of depression, QoL and visuospatial function.

The genetic architecture of PD is increasingly recognized as heterogeneous, with numerous risk variants contributing to disease susceptibility and distinct molecular pathways potentially influencing clinical heterogeneity and disease progression (Horoufi & Zaeifi, 2025; Kim et al., 2024). This complexity suggests that additional genetic variants beyond established PD risk loci may function as modifiers of specific clinical trajectories. In this context, TMEM106B has emerged as a potential candidate modifier. *TMEM106B* variants were first identified as forceful risk factors for developing FTLD (Van Deerlin et al., 2010; van der Zee et al., 2011). Furthermore, the effects of *TMEM106B* SNPs, particularly rs1990622 and rs3173615, on disease progression and clinical-pathological phenotypes have been extensively investigated in FTLD (Tropea et al., 2019; Limorenko & Lashuel, 2021; Pottier et al., 2018; Vandebergh et al., 2024). Growing evidence indicates that TMEM106B is involved in the pathogenesis of NDs beyond FTLD (Hu et al., 2021; Mao et al., 2021). In particular, a recent astounding finding that a previously unknown amyloid fibril deposited in multiple NDs, including PD, was formed by TMEM106B, further strengthens the nonnegligible role of TMEM106B in NDs (Schweighauser et al., 2022; Chang et al., 2022; Fan et al., 2022). Nonetheless, to date only three studies have explored the variants of *TMEM106B* and their relationships with clinical manifestations in PD patients (Tropea et al., 2019; Hu et al., 2017; Zhang et al., 2025), which means the investigation into the role of TMEM106B in PD is still in its infancy. Motor symptoms, including resting tremor, bradykinesia and rigidity, are cardinal manifestations of PD. A cross-sectional study reported that the minor allele of *TMEM106B* rs1990622 was associated with an increased risk of initially manifesting as rigidity/bradykinesia in PD patients (Hu et al., 2017). However, in our PD cohort, no relationship was observed between the *TMEM106B* rs3173615 genotypes and baseline motor symptoms, nor was there any effect of rs3173615 on the longitudinal progression of motor symptoms assessed by both H&Y and UPDRS-III. These inconsistent results suggest the potential different roles that different SNPs of *TMEM106B* played in PD. Depression is one of the most common NMS in PD. Both proteome- and transcriptome-wide association studies identified that *TMEM106B* is one of the candidate causal genes for depression (Deng et al., 2022). Notably, this study found for the first time that PD patients classified into GC group exhibit a faster progression in depression as measured by the GDS. This association suggests that depression trajectory may represent one phenotypic dimension through which *TMEM106B* genotypes modify PD progression. However, given the observational nature of this study, these findings should be interpreted as genotype–phenotype associations rather than direct evidence of causal mechanisms.

The relationships between the genetic variations of *TMEM106B*, especially SNP rs1990622, and cognitive function of an array of NDs were relatively well-explored during the past decade. For FTLD patients, carriers with heterozygotes of SNP rs1990622 showed a more rapid decline in cognition (Tropea et al., 2019). Besides, the major allele of rs1990622 was found to be related to poor cognitive function in ALS patients (Vass et al., 2011), while another study reported that the minor allele of rs1990622 increased cognitive impairment (Manini et al., 2022). Furthermore, a previous study suggested that PD patients carrying major allele of rs1990622 showed faster decrease in global cognitive functions evaluated by both MMSE and Mattis Dementia Rating Scale-2 (Tropea et al., 2019). Although no significant association was observed between rs3173615 genotypes and longitudinal changes in MMSE scores, our study found that both the GG and GC groups exhibited a greater decline in visuospatial function assessed by the CDT compared with the CC group. In addition, the GC group showed a faster decline in visuospatial function assessed by the CFT, while a similar trend was observed in the GG group but did not reach statistical significance.

As one of the most important outcome indicators for disease management, the assessment of QoL in PD patients is imperative. Given the incurable status of PD currently, the amelioration or preservation of QoL is a crucial objective of intervention and therapy for PD. The clinical contributors to and predictors of QoL in PD have been extensively studied (Fan et al., 2020; Fan et al., 2021). Nonetheless, few studies have investigated the effects of SNP on QoL in PD patients. In the present study, QoL deteriorated more rapidly in the GC group than in the CC group over the follow-up period. In addition, the GG group showed a similar trend toward a faster increase in PDQ-39 scores compared with the CC group, although the association did not reach statistical significance (*β* = 1.6158, *p* = 0.0553). These findings suggest that rs3173615 genotypes may influence the longitudinal deterioration of QoL in PD. Further studies are needed to elucidate the underlying mechanisms. Considering that depression is a critical determinant of QoL in PD (Fan et al., 2020; Fan et al., 2021), the influence that rs3173615 has on QoL may partially result from its effects on depression. Of note, rs3173615 and rs1990622 are in near-complete linkage disequilibrium in East Asian populations (1,000 Genomes EAS: D′ = 0.97, *r*^2^ = 0.94), with the G allele of rs3173615 highly correlated with the G allele of rs1990622. As conditional analyses were not performed in the present study, the independent contributions of these variants cannot be determined and should be addressed in future studies with larger cohorts.

The rs3173615 variant results in a nonsynonymous substitution (p.Thr185Ser) in TMEM106B. Previous studies have shown that the TMEM106B protein containing Thr185 is degraded more slowly than the protein containing Ser185. Consistently, overexpression of the Ser185 variant resulted in lower TMEM106B protein levels compared with the Thr185 variant, suggesting that the p.Thr185Ser substitution may regulate TMEM106B protein stability and expression (Nicholson et al., 2013). In addition, evidence suggests that the p.Thr185Ser variant may confer neuroprotective properties in aging and NDs by influencing neuronal development, as it has been associated with enhanced synaptic gene expression and increased neuronal complexity *in vivo* (Nguyen et al., 2023). It has also been reported that rs3173615 genotypes correlate with TMEM106B fibrillar aggregate burden in human brain. Specifically, the burden of pathological TMEM106B aggregates deposited in the brains of NDs patients carrying the GG and GC genotype was higher than that in patients carrying the CC genotype (Cristina et al., 2023). These findings suggest that rs3173615 may be associated with differences in TMEM106B protein homeostasis and aggregation propensity; however, whether these molecular associations contribute to the clinical phenotypes observed in PD remains to be determined.

Beyond its potential involvement in protein aggregation, TMEM106B may influence PD progression through its established role in lysosomal homeostasis. Lysosomal dysfunction has emerged as an important pathogenic mechanism in PD, contributing to impaired degradation of misfolded proteins, including *α*-syn, and promoting neurodegeneration (Shimizu et al., 2026; Gan-Or et al., 2015). TMEM106B encodes a lysosomal transmembrane protein involved in lysosomal morphology, trafficking, and function (Takahashi & Strittmatter, 2025). Recent discoveries of TMEM106B fibrillar pathology in NDs have further provided new insights into its potential involvement in protein aggregation-related mechanisms (Schweighauser et al., 2022; Chang et al., 2022; Fan et al., 2022; Takahashi & Strittmatter, 2025). Given previous associations between *TMEM106B* genetic variation and cognitive and neuropsychiatric phenotypes (Tropea et al., 2019; Vandebergh et al., 2024; Deng et al., 2022), altered TMEM106B biology may contribute to the progression of NMS in PD. However, whether rs3173615 directly influences lysosomal function, TMEM106B aggregation, or downstream neurodegenerative pathways remains unclear and requires further functional investigation.

The primary strength of this study lies in its relatively systematic exploration and elucidation of the effects of rs3173615, the only coding SNP of *TMEM106B*, on clinical manifestations in a longitudinally followed PD cohort. Notwithstanding, several limitations of this study should be acknowledged. First, this is a single-center study conducted at Huashan Hospital; thus, potential selection bias in subject recruitment could not be fully precluded. Therefore, multi-center research is urgently needed to further confirm these findings to enhance the credibility of the conclusions in the study. Second, the follow-up duration in this study was relatively short, and varied among PD patients, with a mean (SD) of 3.99 (1.12) years. Nevertheless, these follow-up times were sufficient to detect disease progression. Besides, the adoption of LMM for analysis allowed for optimal utilization of data with heterogeneous follow-up times. Third, stratification by rs3173615 genotype resulted in relatively small subgroup sample sizes, particularly for the CC group (n = 31), reflecting allele frequency distribution in East Asian populations. This may have reduced statistical power and the precision of effect estimates. Studies with larger sample sizes are needed to validate these findings. Fourth, all our PD patients are of Asian descent. Therefore, further studies are warranted to determine whether these findings can be replicated across other racial populations, given the differences in frequency distribution of the alleles of rs3173615 among different races (Fan et al., 2023). Finally, formal correction for multiple testing was not applied due to the exploratory nature of the analyses and the correlation among clinical phenotypes. Therefore, the observed associations should be interpreted as hypothesis-generating findings and require validation in independent cohorts.

## Conclusion

In conclusion, we mainly investigated the effects of *TMEM106B* SNP rs3173615 on disease progression in PD, and revealed that rs3173615 is a genetic modifier of the longitudinal deterioration in depression, visuospatial function and QoL. These findings suggest that TMEM106B is involved in the pathogenesis and progression of PD, and indicate the potential benefits of interventions and therapies targeting TMEM106B in PD.

## Data Availability

The data analyzed in this study is subject to the following licenses/restrictions: Restrictions: The dataset is available upon request from the corresponding authors and is not publicly accessible. Data may only be used for research integrity verification or with explicit permission. Redistribution and commercial use are prohibited. Requests to access these datasets should be directed to Yimin Sun, ys2504@sina.com; Jian Wang, wangjian_hs@fudan.edu.cn; Yun Fan, fanyun_hs@fudan.edu.cn.
