# Supplementary material for: Effects of transmembrane protein 106B genetic variations on disease progression in Parkinson’s disease
Source: Front Aging Neurosci. 2026 Jul 23;18:1873947. doi: 10.3389/fnagi.2026.1873947 (PMC13442443; doi:10.3389/fnagi.2026.1873947)
Supplement: Supplementary file 1 [file Data_Sheet_1.docx]

**Supplementary information**

**Effects of transmembrane protein 106B genetic variations on disease progression in Parkinson’s disease**

Wanbing Zhao^1#^, Xiaoniu Liang^1#^, Wenbo Yu^1^, Jianjun Wu^1^, Yimin Sun^1*^, Jian Wang^1*^, Yun Fan^1*^

^1^Department of Neurology and National Research Center for Aging and Medicine & National Center for Neurological Disorders, State Key Laboratory of Medical Neurobiology, Huashan Hospital, Fudan University, Shanghai, 200040, China

^#^These authors have contributed equally to this work.

**Table S1. Relationship with clinical phenotypes at baseline compared with the CC group.**

|  | GG | |  | GC | |
| --- | --- | --- | --- | --- | --- |
|  | β (95% CI) | *p* value |  | β (95% CI) | *p* value |
| PDQ-39 | -1.9302 (-15.4922, 11.6318) | 0.7785 |  | -2.7787 (-15.5129, 9.9556) | 0.6664 |
| UPDRS-III | -1.3615 (-9.2008, 6.4778) | 0.7313 |  | -1.6261 (-8.8782, 5.6259) | 0.6576 |
| GDS | -1.2608 (-5.1871, 2.6655) | 0.5260 |  | -0.2024 (-3.8717, 3.4670) | 0.9132 |
| BDI | -3.1524 (-8.5071, 2.2024) | 0.2460 |  | -3.2882 (-8.3135, 1.7370) | 0.1975 |
| NMSS | 1.8365 (-1.4007, 5.0737) | 0.2635 |  | 0.2932 (-2.7448, 3.3312) | 0.8487 |
| RBDSQ | 0.0689 (-1.7300, 1.8678) | 0.9396 |  | -0.5792 (-2.2597, 1.1014) | 0.4960 |
| SSST-12 | 0.2202 (-1.400, 1.8407) | 0.7876 |  | -0.2332 (-1.7336, 1.2672) | 0.7580 |
| MMSE | -0.8579 (-2.8413, 1.1256) | 0.3932 |  | -0.0190(-1.8702, 1.8322) | 0.9838 |
| AVLT-Delay recall | 1.1296 (-0.2430, 2.5023) | 0.1057 |  | 0.4875 (-0.7683, 1.7433) | 0.4431 |
| AVLT-T | 3.1840 (-1.9149, 8.2828) | 0.2183 |  | 1.7465 (-2.9183, 6.4113) | 0.4594 |
| CFT-delay recall | 1.3525 (-2.8207, 5.5257) | 0.5218 |  | 2.0621 (-1.7570, 5.8811) | 0.2867 |
| CWT-C TIME(s) | 2.6500 (-15.7266, 21.0266) | 0.7753 |  | -0.4388 (-17.2568, 16.3792) | 0.9588 |
| CWT-C right | -0.1766 (-4.0335, 3.6802) | 0.9278 |  | -0.4406 (-3.9703, 3.0892) | 0.8048 |
| TMT-B(s) | 3.3250 (-41.6127, 48.2628) | 0.8835 |  | 26.7305 (-14.1725, 67.6336) | 0.1976 |
| BNT | 0.7434 (-1.6906, 3.1774) | 0.5461 |  | 0.1674 (-2.0639, 2.3987) | 0.8820 |
| AFT | 0.8478 (-1.5016, 3.1971) | 0.4758 |  | 0.3621 (-1.7860, 2.5102) | 0.7388 |
| CFT | -0.1054 (-5.4536, 5.2427) | 0.9689 |  | -0.1286 (-4.9947, 4.7376) | 0.9583 |
| CDT | 2.0177 (-2.2766, 6.3120) | 0.3531 |  | 1.6922 (-2.1280, 5.5123) | 0.3812 |
| SDMT | 4.0155 (-2.4157, 10.4467) | 0.2183 |  | 1.6348 (-4.2470, 7.5166) | 0.5825 |
| TMT-A(s) | -1.7504 (-31.5233, 28.0225) | 0.9074 |  | 10.7630 (-16.3573, 37.8832) | 0.4329 |

AFT, Animal Fluency Test; AVLT, Auditory Verbal Learning Test; β, standardized beta coefficient; BDI, Beck Depression Inventory; BNT, Boston Naming Test; CDT, Clock Drawing Test; CFT, Rey-Osterrieth Complex Figure Test; CI, confidence interval; CWT, Stroop Color-Word Test; GDS, Geriatric Depression Rating Scale; MMSE, Mini Mental State Examination; NMSS, Non-Motor Symptoms Scale; PDQ-39, 39-item Parkinson’s disease questionnaire; RBDSQ, Rapid-Eye-Movement Sleep Behavior Disorder Screening Questionnaire; SSST-12, Sniffin’ Sticks screening 12 test; SDMT, Symbol Digit Modality Test; TMT, Trail Making Test; UPDRS-III, Unified Parkinson’s Disease Rating Scale part III.

**Table S2. Sensitivity analysis of *TMEM106B* rs3173615 under alternative genetic models for ESS.**

|  | Genetic Model | β (95% CI) | *p* value |
| --- | --- | --- | --- |
| ESS | Dominant (GC+CC vs GG) | 0.2838 (-1.5722, 2.1399) | 0.7624 |
|  | Recessive (CC vs GG+GC) | 3.4067 (1.0474, 5.7659) | 0.0050** |
|  | Overdominant (GC vs GG+CC) | -1.4766 (-3.1948, 0.2416) | 0.0914 |
|  | Additive (GG=0, GC=1, CC=2) | 1.1155 (-0.1755, 2.4064) | 0.0896 |

β, beta coefficient; CI, confidence interval; ESS, Epworth Sleepiness Score.

**p* < 0.05, ***p* < 0.01.

**Table S3. Effects of *TMEM106B* SNPs on longitudinal clinical phenotypes’ progression in PD patients.**

|  | GG | | |  | GC | | |  | CC | | |
| --- | --- | --- | --- | --- | --- | --- | --- | --- | --- | --- | --- |
|  | β | SE | *p* value |  | β | SE | *p* value |  | β | SE | *p* value |
| CDT | -0.3692 | 0.1507 | 0.0143* |  | -0.4090 | 0.1205 | 0.0007*** |  | 0.4997 | 0.2353 | 0.0337* |
| CFT | -0.4441 | 0.1896 | 0.0192* |  | -0.6368 | 0.1553 | 0.0000**** |  | 0.1685 | 0.3143 | 0.5918 |
| GDS | 0.4396 | 0.13046 | 0.0008*** |  | 0.5028 | 0.1129 | 0.0000**** |  | -0.0218 | 0.2095 | 0.9171 |
| PDQ-39 | 2.8637 | 0.4594 | 0.0000**** |  | 3.0000 | 0.3865 | 0.0000**** |  | 1.2528 | 0.7397 | 0.0903 |

β, beta coefficient; CDT, Clock Drawing Test; CFT, Rey-Osterrieth Complex Figure Test; GDS, Geriatric Depression Rating Scale; PDQ-39, 39-item Parkinson’s disease questionnaire; SE, standard error.

**p* < 0.05, ****p* < 0.001, *****p* < 0.0001.

**Table S4. Estimates for change in clinical phenotypes trajectory compared with the CC group.**

|  | GG | |  | GC | |
| --- | --- | --- | --- | --- | --- |
|  | β (95% CI) | *p* value |  | β (95% CI) | *p* value |
| UPDRS-III | 0.9330 (-0.0131, 1.8790) | 0.0532 |  | 0.5892 (-0.3091, 1.4875) | 0.1974 |
| BDI | 0.3657 (-0.2548, 0.9862) | 0.2469 |  | 0.5238 (-0.0693, 1.1170) | 0.0832 |
| NMSS | -0.1916 (-0.7008, 0.3177) | 0.4595 |  | -0.0081 (-0.4956, 0.4794) | 0.9739 |
| ESS | 0.0335 (-0.3072, 0.3742) | 0.8464 |  | 0.2012 (-0.1265, 0.5290) | 0.2277 |
| RBDSQ | -0.0255 (-0.2683, 0.2172) | 0.8360 |  | 0.1451 (-0.0876, 0.3777) | 0.2205 |
| SSST-12 | 0.0629 (-0.1098, 0.2355) | 0.4735 |  | 0.0730 (-0.0933, 0.2391) | 0.3878 |
| MMSE | -0.0449 (-0.3160, 0.2263) | 0.7440 |  | -0.0401 (-0.2965, 0.2163) | 0.7576 |
| AVLT-Delay recall | -0.0104 (-0.2277, 0.2070) | 0.9250 |  | -0.0304 (-0.2376, 0.1769) | 0.7725 |
| AVLT-T | -0.1314 (-0.9110, 0.6483) | 0.7394 |  | -0.1651 (-0.9112, 0.5811) | 0.6623 |
| CFT-delay recall | -0.1325 (-0.7658, 0.5009) | 0.6795 |  | -0.1695 (-0.7730, 0.4340) | 0.5792 |
| CWT-C time(s) | 1.0869 (-2.9205, 5.0943) | 0.5923 |  | 2.4153 (-1.3709, 6.2015) | 0.2090 |
| CWT-C right | -0.6572 (-1.4137, 0.0994) | 0.0880 |  | -0.4437 (-1.1579, 0.2705) | 0.2211 |
| TMT-B(s) | -1.2571 (-8.2621, 5.7478) | 0.7228 |  | 0.7817 (-5.7640, 7.3275) | 0.8134 |
| BNT | -0.0163 (-0.3233, 0.2907) | 0.9164 |  | -0.1408 (-0.4312, 0.1496) | 0.3392 |
| AFT | 0.2953 (-0.0483, 0.6389) | 0.0914 |  | 0.2075 (-0.1178, 0.5328) | 0.2090 |
| SDMT | 0.8146 (-0.2381, 1.8673) | 0.1281 |  | 0.5288 (-0.4685, 1.5261) | 0.2958 |
| TMT-A (s) | 1.9153 (-3.0458, 6.8765) | 0.4461 |  | 4.4928 (-0.1820, 9.1675) | 0.0595 |

AFT, Animal Fluency Test; AVLT, Auditory Verbal Learning Test; β, beta coefficient; BDI, Beck Depression Inventory; BNT, Boston Naming Test; CFT, Rey-Osterrieth Complex Figure Test; CI, confidence interval; CWT, Stroop Color-Word Test; ESS, Epworth Sleepiness Score; MMSE, Mini Mental State Examination; NMSS, Non-Motor Symptoms Scale; RBDSQ, Rapid-Eye-Movement Sleep Behavior Disorder Screening Questionnaire; SSST-12, Sniffin’ Sticks screening 12 test; SDMT, Symbol Digit Modality Test; TMT, Trail Making Test; UPDRS-III, Unified Parkinson’s Disease Rating Scale part III.

**Table S5. Sensitivity analysis of *TMEM106B* rs3173615 under alternative genetic models for longitudinal outcomes.**

|  | Genetic Model | β (95% CI) | *p* value |
| --- | --- | --- | --- |
| CDT | Dominant (GC+CC vs GG) | 0.1166 (-0.2432, 0.4763) | 0.5225 |
|  | Recessive (CC vs GG+GC) | 0.8897 (0.3977, 1.3816) | 0.0005*** |
|  | Overdominant (GC vs GG+CC) | -0.2774 (-0.6141, 0.0593) | 0.1055 |
|  | Additive (GG=0, GC=1, CC=2) | 0.2959 (0.0374, 0.5544) | 0.0252* |
| CFT | Dominant (GC+CC vs GG) | -0.0648 (-0.5098, 0.3802) | 0.7738 |
|  | Recessive (CC vs GG+GC) | 0.7180 (0.0728, 1.3631) | 0.0295* |
|  | Overdominant (GC vs GG+CC) | -0.3496 (-0.7715, 0.0724) | 0.1036 |
|  | Additive (GG=0, GC=1, CC=2) | 0.1497 (-0.1783, 0.4776) | 0.3682 |
| GDS | Dominant (GC+CC vs GG) | -0.0462 (-0.3555, 0.2630) | 0.7687 |
|  | Recessive (CC vs GG+GC) | -0.5123 (-0.9496, -0.0750) | 0.0219* |
|  | Overdominant (GC vs GG+CC) | 0.1904 (-0.1030, 0.4838) | 0.2024 |
|  | Additive (GG=0, GC=1, CC=2) | -0.1621 (-0.3870, 0.0628) | 0.1570 |
| PDQ-39 | Dominant (GC+CC vs GG) | -0.2268 (-1.3286, 0.8749) | 0.6855 |
|  | Recessive (CC vs GG+GC) | -1.7022 (-3.2188, -0.1856) | 0.0280* |
|  | Overdominant (GC vs GG+CC) | 0.5680 (-0.4621, 1.5980) | 0.2785 |
|  | Additive (GG=0, GC=1, CC=2) | -0.5579 (-1.3550, 0.2392) | 0.1693 |

β, beta coefficient; CDT, Clock Drawing Test; CFT, Rey-Osterrieth Complex Figure Test; CI, confidence interval; GDS, Geriatric Depression Rating Scale; PDQ-39, 39-item Parkinson’s disease questionnaire.

**p* < 0.05, ****p* < 0.001.
